# The Role of Interleukin-10 in Mediating the Effect of Immune Challenge on Mouse Gonadotropin-Releasing Hormone Neurons *In Vivo*

**DOI:** 10.1523/ENEURO.0211-18.2018

**Published:** 2018-10-15

**Authors:** Klaudia Barabás, Zsuzsanna Barad, Ádám Dénes, Janardhan P. Bhattarai, Seong-Kyu Han, Endre Kiss, Gabriella Sármay, István M. Ábrahám

**Affiliations:** 1 MTA NAP-B Molecular Neuroendocrinology Research Group, Institute of Physiology, Medical School, Centre for Neuroscience, Szentágothai Research Institute, University of Pécs, 7624 Pécs, Hungary; 2 Centre for Neuroendocrinology and Department of Physiology, Otago School of Medical Sciences, University of Otago, Dunedin 9054, New Zealand; 3 Department of Oral Physiololgy, School of Dentistry and Institute of Oral Bioscience, Chonbuk National University, Jeonju 567, Republic of Korea; 4 Department of Immunology, Eötvös Loránd University, Budapest, Hungary; 5 Laboratory of Neuroimmunology, Hungarian Academy of Sciences, Institute of Experimental Medicine, Budapest 1119, Hungary

**Keywords:** ERK1/2, GnRH neurons, IL-10, immune challenge, T-cell-dependent B-cell response

## Abstract

Immune challenge alters neural functioning via cytokine production. Inflammation has profound impact on the central regulation of fertility, but the mechanisms involved are not clearly defined. The anti-inflammatory cytokine interleukin (IL)-10 is responsible for balancing the immune response in the brain. To examine whether IL-10 has an effect on the function of the gonadotropin-releasing hormone (GnRH) neurons, we first examined the effect of immune responses with distinct cytokine profiles, such as the T cell-dependent (TD) and T cell-independent (TI) B-cell response. We investigated the effect of the TD and TI immune responses on ERK1/2 phosphorylation in GnRH neurons by administering fluorescein isothiocyanate/keyhole limpet hemocyanin (KLH-FITC) or dextran-FITC to female mice. Although dextran-FITC had no effect, KLH-FITC induced ERK1/2 phosphorylation in GnRH neurons after 6 d. KLH-FITC treatment increased the levels of IL-10 in the hypothalamus (HYP), but this treatment did not cause lymphocyte infiltration or an increase in the levels of proinflammatory cytokines. In IL-10 knock-out (KO) mice, KLH-FITC-induced ERK1/2 phosphorylation in the GnRH neurons was absent. We also showed that in IL-10 KO mice, the estrous cycle was disrupted. Perforated patch-clamp recordings from GnRH-GFP neurons, IL-10 immunohistochemistry, and in vitro experiments on acute brain slices revealed that IL-10 can directly alter GnRH neuron firing and induce ERK1/2 phosphorylation. These observations demonstrate that IL-10 plays a role in influencing signaling of GnRH neurons in the TD immune response. These results also provide the first evidence that IL-10 can directly alter the function of GnRH neurons and may help the maintenance of the integrity of the estrous cycle.

## Significance Statement

The anti-inflammatory cytokine interleukin (IL)-10 plays a role in maintaining a balanced immune response in the brain. Although clinical studies have demonstrated that IL-10 influences fertility, the impact of IL-10 on gonadotropin-releasing hormone (GnRH) neurons is unknown. Our results demonstrate that T cell-dependent (TD) immune challenge induces ERK1/2 phosphorylation in GnRH neurons via IL-10. We provide the first evidence that IL-10 directly regulates the firing and signaling of GnRH neurons. Our results indicate that IL-10-induced ERK1/2 phosphorylation in GnRH neurons might be associated with the maintenance of the estrous cycle in bacterial/viral infection.

## Introduction

Immune challenge is often associated with perturbances in brain function that disrupt homeostatic control mechanisms such as the neuroendocrine balance by modulating neuronal activity in the brain (ThyagaRajan and Priyanka, 2012). Interleukin (IL)-10 is an essential anti-inflammatory cytokine that regulates peripheral immune cells during excessive inflammation and it also maintains the balance of the immune response in the brain (Lobo-Silva et al., 2016; Norden et al., 2016). IL-10 is released from several cell types, including astrocytes and microglia, after immune challenge and brain injury (Schroeter and Jander, 2005; Rodney et al., 2018). Although there is growing evidence that the role of IL-10 in neurons is to induce anti-apoptotic effects during neurodegenerative diseases and brain injuries (Xin et al., 2011; Lobo-Silva et al., 2016; Lan et al., 2017), little if any attention has been given to the potential regulatory role of IL-10 in neuronal functions that regulate fertility.

Basal forebrain neurons that synthesize and secrete gonadotropin-releasing hormone (GnRH) are the final output cells of a complex neuronal network that regulates fertility in all mammalian species (Herbison, 2016). In the hypothalamic-pituitary-gonadal (HPG) axis, periodic release of GnRH into the hypophyseal portal system via the median eminence induces a pulsatile pattern of pituitary gonadotrophs regulating the gonadal function during the estrous cycle. Although clinical data show that IL-10 plays a key role in regulating fertility (Wegmann et al., 1993; van Dunné et al., 2006; Cheng and Sharma, 2015), and it has been demonstrated that various cytokines have the capacity to act on GnRH neurons (Bouret et al., 2004; Igaz et al., 2006; Kuwahara-Otani et al., 2017; Sarchielli et al., 2017), the effect of IL-10 on GnRH neurons remains unknown.

As secreted cytokines act via different intracellular signaling pathways, such as ERK1/2, to fine-tune neuronal functions (Kim et al., 2013; Sticozzi et al., 2013), we have used ERK1/2 phosphorylation as an index of changes in intracellular alterations in GnRH neurons. We applied multiple *in vivo* and *in vitro* techniques to explore the effect of IL-10 on GnRH neurons. Our findings demonstrate that IL-10 alters the intracellular signaling of GnRH in the immune response and maintains the integrity of the estrous cycle. These studies provide the first evidence for IL-10 as a regulator of the functions of GnRH neurons.

## Materials and Methods

### Animals

Adult female wild-type (WT; C57BL6/J, 42–56 d old), GnRH-GFP (40–54 d old; Spergel et al., 1999), and IL-10 knock-out (KO; 129(B6)-Il10tm1Cgn/J, The Jackson Laboratory) mice were bred and housed under 12/12 h light/dark cycle conditions and supplied with food and water ad libitum. Breeding and all the experiments were approved by local animal ethic committees (protocol number: 96/07).

### Animal treatments

Immunization of animals was performed as described previously (Adori et al., 2010). Briefly, for immunization, intact WT (C57BL/6) or IL-10 KO (129(B6)-Il10^tm1Cgn^/J) female mice and their WT littermates (129(B6)/J) were treated with 200 μg of T cell-dependent (TD) antigen [keyhole limpet hemocyanin/fluorescein isothiocyanate (KLH-FITC); KLH: Sigma, FITC: Invitrogen] or 200 μg of T cell-independent (TI) antigen (dextran-FITC, Fluka BioChemika). The TD antigen, KLH-FITC (in 50 μl of PBS) mixed with 250 μl of complete Freund’s adjuvant (CFA) was administered subcutaneously into the tail base as the KLH-FITC injection is more effective when introduced subcutaneously since in the TD immune response the lymph nodes and the dendritic cells play the critical role (Murphy and Weaver, 2017). In case of the TI response, the peritoneal B cells give a robust response (Murphy and Weaver, 2017); therefore, the dextran-FITC (in PBS) was administered intraperitoneally. The control mice for the KLH-FITC and dextran-FITC treatments received CFA and PBS, respectively. Vaginal smears were taken before KLH-FITC, dextran-FITC, CFA, or PBS injection, and animals in the diestrus stage were selected for each immunization.

Immunized and control animals were deeply anesthetized by an overdose of Avertin at different time points (6 h and 3, 6, and 12 d) after treatment. Blood was taken for ELISA, and the mice were transcardially perfused with ice-cold physiologic saline for flow cytometry or 4% paraformaldehyde (PFA) for immunohistochemistry. C57BL6/J mice, IL-10 KO mice and their WT littermates were perfused with 4% PFA on day 6 after immunization. For immunohistochemistry, brains were removed from the skulls, perfused with 4% PFA, postfixed for 2 h at 4°C, and placed in 0.1 M Tris-buffered saline (TBS) solution containing 30% sucrose overnight at 4°C. Coronal sections (30-μm thick) were cut on a freezing microtome, and four sets of sections were collected in TBS.

For Western blot studies, KLH-FITC-injected and control mice were deeply anesthetized by an overdose of Avertin and decapitated 6 d after immunization. Then, the brain regions were micro-dissected or micro-punched bilaterally (where appropriate) as described previously (Palkovits, 1986; Sárvári et al., 2009). The following brain regions were micro-punched on the basis of Franklin and Paxinos mouse brain atlas: medial septum (MS; plate 21–28), striatum (S; plate 21–36), thalamus (T; plate 36–48), and somatosensory cortex (C; plate 21–46). The hypothalamus (HYP), hippocampus (HC), and pituitary (P) were micro-dissected. Brain tissue was stored at -80°C until sample preparation.

In separate experiments, 5-mg/kg indomethacin (Fluka, Sigma; diluted in 2.5% ethanol with 0.9% NaCl) was administered intraperitoneally to mice (*n* = 5) 30 min (Elmorsi et al., 2013) before KLH-FITC immunization and then daily until the sixth day, when the mice were deeply anesthetized by an overdose of Avertin and perfused with 4% PFA. Control animals received the vehicle of indomethacin.

### Evaluation of the estrous cycle

To evaluate the effect of IL-10 on the estrous cycle, vaginal smears were taken daily (10 A.M.) from IL-10 KO mice and WT littermates for a period of five weeks. Vaginal material was stained by a 0.1% aqueous solution of methylene blue and examined under a microscope to evaluate the stage of the estrous cycle.

### *In vitro* experiments on acute brain slices

In this study, we used an acute brain slice preparation protocol that has been described previously (Cheong et al., 2012). Briefly, adult female mice were decapitated, and their brains were rapidly removed and maintained in oxygenated, ice-cold, artificial CSF (ACSF) cutting solution containing 118 mM NaCl, 3 mM KCl, 0.5 mM CaCl_2_, 6 mM MgCl_2_, 11 mM D-glucose, 10 mM HEPES, and 25 mM NaHCO_3_ (pH 7.4 when bubbled with carbogen; 95% O_2_, 5% CO_2_). Coronal slices (250-μm thick) were cut on a vibratome (Leica VT-1000; Leica) before being preincubated at 30°C for 30 min in oxygenated normal recording ACSF saturated with 95% O_2_ and 5% CO_2_ containing the following: 118 mM NaCl, 3 mM KCl, 2.5 mM CaCl_2_, 1.2 mM MgCl_2_, 11 mM D-glucose, 10 mM HEPES, and 25 mM NaHCO_3_. To investigate the effect of the blockade of action potential-dependent neurotransmitter release and GABA_A_/glutamate receptors on the IL-10-induced effects, slices were transferred into ACSF-containing blocking mixture (BM) with 0.5 μM tetrodotoxin (TTX; Alomone Labs), 100 μM picrotoxin (Sigma), 20 μM 6-cyano-7-nitroquinoxaline-2,3-dione (CNQX; Tocris Bioscience), and 20 μM DL-2-amino-5-phosphonopentanoic acid (AP5; Tocris Bioscience). The concentrations of the BM used in this study have been reported to be effective in brain slice experiments (Lee et al., 2010; Cheong et al., 2012). After 30 min of incubation, 2 ng/ml IL-10 was added to the slice incubation medium for 15 min. Slices were then washed and placed overnight at 4°C in 4% PFA before being placed in TBS containing 30% sucrose for 3 h. Four sets of 30-μm-thick sections were then cut in the coronal plane on a freezing microtome, collected in TBS, and examined for phosphorylated ERK1/2 (pERK1/2), ERK1/2, and GnRH immunoreactivity.

### Immunohistochemistry

Dual-label immunohistochemistry was performed to detect pERK1/2 or ERK1/2 immunoreactivity within the GnRH neurons as described previously (Cheong et al., 2012). Briefly, brain sections of control and immunized animals were incubated with either monoclonal rabbit anti-p44/42 MAP kinase antibody (ERK1/2; 1:1000; Cell Signaling Technology) or rabbit anti-phospho-p44/42 MAP kinase (pERK1/2; 1:1000; Cell Signaling Technology) antibody in TBS for 48 h at 4°C, which was followed by incubation with polyclonal sheep anti-mouse GnRH-specific antibody (1:10000, gift from Alain Caraty, France) for an additional 48 h at 4°C. This step was followed by incubation for 2 h at room temperature (RT) in Cy5-conjugated anti-rabbit antibody (1:200, The Jackson Laboratory) or biotinylated anti-sheep IgG (1:200, Vector Laboratories) and streptavidin-Alexa Fluor 488 (1:1000, Invitrogen), as appropriate. The pERK1/2 antibody detects endogenous levels of p44 and p42 MAP kinase (pERK1 and pERK2) when phosphorylated either individually or simultaneously at Thr202 and Tyr204 of ERK1 as well as Thr185 and Tyr187 of ERK2.

The detection of IL-10R in GnRH neurons was accomplished by biotin tyramide-amplified dual-label immunohistochemistry. Brain sections of C57BL/6 female mice were pretreated with 3% H_2_O_2_/TBS for 15 min and blocked with 5% horse serum (HS)/0.1% Triton X-100/TBS for 30 min at RT, then incubated with polyclonal rabbit anti-mouse IL-10 receptor subunit α (IL-10RA, 1:500, MyBiosource) antibody for 48 h at 4°C. Sections then were incubated in biotinylated donkey anti-rabbit antibody (1:200, The Jackson Laboratory, 2 h, RT), followed by avidin-biotin-HRP complex (ABC, The Jackson Laboratory, 2 h, RT) and Alexa Fluor 594 NHS Ester (Invitrogen) in 0.01% H_2_O_2_/TBS for 15 min at RT. The GnRH signal was detected by incubation with GA02 antibody (1:1000, a gift from G. Anderson, 48 h, 4°C), followed by biotinylated donkey anti-guinea pig antibody (1:2000, The Jackson Laboratory, 2 h, RT) and then with Avidin Alexa 488 (1:2000, The Jackson Laboratory, 2 h, RT).

The specificities of the primary antibodies of ERK1/2, pERK1/2, and GnRH have been reported previously in rodent species (Abrahám et al., 2004; Barabás et al., 2006). The specificity of IL-10RA antibody have been validated by preadsorption of the primary antibody with its corresponding fusion protein. The omission of primary antibodies resulted in a complete absence of the corresponding immunoreactivity in these studies.

### Image analysis

A Zeiss (Zeiss LSM 510, Carl Zeiss Jena GmbH) confocal laser scanning system equipped with a multi-argon laser, and helium-neon lasers was used to detect ERK1/2, pERK1/2, and IL-10RA immunoreactivity within GnRH neurons. An argon laser was used to excite Alexa Fluor 488-labeled GnRH neurons at a wavelength of 488 nm, and a helium-neon laser was used to excite Cy5-labeled ERK1/2- or pERK1/2-containing neurons at a wavelength of 649 nm and Alexa Fluor 594-labeled IL-10RA at a wavelength of 594 nm. The diameter of the pinhole aperture was 80 μm, resulting in a 1.5-μm optical thickness. Quantitative evaluation of the double-labeled tissue was performed by an investigator blinded to the experimental groupings. Analysis of the double-labeled tissue was conducted by manually counting the number of single- and dual-labeled GnRH neurons. GnRH neurons located in the MS and preoptic area (POA) and at the level of the anterior hypothalamic area (AH) were examined. Using the Paxinos and Franklin (2001) mouse brain atlas, three sections from each region were selected for analysis: MS (plates 23–26), POA (plates 25–28) and AH (plates 34–36). In case of IL-10RA immunohistochemistry, confocal stacks were prepared from GnRH neurons (0.5-µm steps, resolution: 1544 × 1544 pixels) and analyzed using Imaris 8.1.2 image analysis software (Bitplane AG). Convoluted surfaces were created and used to identify the location and number of IL-10RA on GnRH neurons in the three-dimensional space. IL-10RA and GnRH neuron coexpressions were analyzed using three-dimensional reconstruction of the confocal images.

### Western blotting

Micro-dissected brain tissue was homogenized on ice in 0.1 M PBS-0.5% Tween buffer containing protease inhibitors [1 µg/ml; leupeptin, pepstatin A, aprotinin, phenylmethanesulfonyl fluoride (PMSF) and vanadate (20 μl of homogenization buffer/1 mg tissue)]. Homogenates were pelleted at high speed; supernatants were collected and stored at -80°C until further use. Proteins were separated by sodium dodecyl sulfate (SDS) polyacrylamide gel electrophoresis and then electrically transferred to PVDF membranes. The membranes were incubated with rabbit anti-mouse pERK1/2 primary antibody (1:1000; Cell Signaling) or monoclonal rabbit ERK1/2-specific primary antibody (1:1000; Cell Signaling). The membranes were then incubated with HRP-conjugated polyclonal anti-rabbit antibody (1:20,000; Abcam). Bands were visualized on x-ray films using the Supersignal West Pico chemiluminescent substrate (Pierce). The membranes were washed extensively with TBS-0.5% Tween buffer between all the incubation steps. The ERK1/2 activity (relative to pERK1/2/ERK1/2 levels) was calculated by normalizing the level of pERK1/2 to the total ERK1/2. pERK1/2/ERK1/2 data for each group were expressed as ratios of the mean values.

### Isolation of lymphocytes and fluorescence-activated cell sorting (FACS)

KLH-FITC-treated and control mice were deeply anesthetized by an overdose of Avertin at 6 d after immunization, and the animals were perfused with physiologic saline. The brains of four KLH-FITC-injected and four control mice were homogenized separately in HBSS buffer (pH 7.4). Samples were then centrifuged at 1200 rpm for 10 min at 4°C. The pellets were resuspended in HBSS buffer containing 1 mg/ml collagenase (Sigma) and 0.1 mg/ml DNase I (Sigma), and the suspension was incubated for 1 h at 37°C. Then, EDTA was added to each sample to avoid cell aggregation at a final concentration of 20 mM for 5 min. Samples were filtered using a 70-μm mesh and pelleted at 1200 rpm for 10 min at 4°C. To isolate lymphocytes, the Percoll separation method was used. Pellets were resuspended in 7 ml of 30% Percoll (Sigma) and it carefully superposed onto 4 ml of 70% Percoll. The samples were then centrifuged at 2000 rpm for 10 min at 4°C. The cells at the interface of the 30 and 70% Percoll solutions were collected and washed with HBSS buffer. At this stage, cells from the same experimental group were pooled. The live cells collected were counted by the standard Trypan blue method (Louis and Siegel, 2011).

To evaluate the lymphocyte infiltration following KLH-FITC immunization, isolated brain cells were washed with FACS buffer (PBS containing 0.5% FCS and 0.1% sodium azide). K9.361, a mouse anti-mouse FcgammaRII antibody, was used to block Fc receptors (5 min, 4°C). B cells were stained with FITC-conjugated anti-mouse B220 antibody. For T-cell detection, an Alexa Fluor 647-conjugated anti-mouse CD3-specific monoclonal antibody (Caltag) was used (30 min 4°C). Flow cytometric analysis was performed in FACSCalibur (Becton Dickinson). Regions for analysis were set according to the forward scatter (FSC, roughly proportional to the diameter of the cell) and side scatter (SSC, proportional to the granularity) characteristics of lymphocytes from the spleen. A total of 2 × 10^5^ fluorescence-labeled cells were analyzed with CellQuest Pro (Becton Dickinson).

### ELISA

To validate the immunizations, 0.2 ml of blood was collected from the right atrium of each mouse before perfusion with PFA to determine the immunoglobulin levels produced against FITC. Plates were coated with BSA-FITC (overnight, 4°C) and then blocked with 1% non-fat milk powder. Serial dilutions of serum samples were incubated on the plates for 2 h at RT, which was followed by incubation with HRP-conjugated polyclonal anti-rabbit IgM and IgG antibodies (1:2000, Dako). The reaction was visualized with H_2_O_2_ and 3,30,5,50-tetramethyl benzidine and stopped with 2 N H_2_SO_4_. The end product was measured in an ELISA reader at a wavelength of 450 nm.

For determination of brain cytokine levels following immunization, the mice were perfused with ice-cold physiologic saline solution, and the dissected brain tissue, containing the HYP and MS regions, was homogenized in 0.1 M PBS-0.5% Tween buffer containing protease inhibitors. Samples were centrifuged, and IL-1β, TNF-α, and IL-10 concentrations in the supernatant were determined by using a quantitative sandwich enzyme immunoassay kit according to the manufacturer’s protocol (R&D Systems). Briefly, the ELISA plates were coated overnight at 4°C with mouse IL-1β-, TNF-α-, or IL-10-specific antibodies. Plates were washed the following day, blocked, and incubated with the brain lysates at appropriate dilutions for 2 h at RT. After washing, the plates were incubated with cytokine-specific biotinylated detection antibodies for 1 h at RT, followed by incubation with avidin-HRP for an additional 30 min. The reaction was initiated with the kit substrate solution and stopped with 2 N HCl, and the plates were read at 450 nm.

### Single-cell electrophysiology

Brain slices were acutely prepared as described (Bhattarai et al., 2014); 40- to 54-d-old female GnRH-GFP mice in the diestrus stage were decapitated, and the brains were rapidly removed and placed in ice-cold ACSF with the following composition: 126 mM NaCl, 2.5 mM KCl, 2.4 mM CaCl_2_, 1.2 mM MgCl_2_, 11 mM D-glucose, 1.4 mM NaH_2_PO_4_, and 25 mM NaHCO_3_ (pH 7.4 when bubbled with 95% O_2_ and 5% CO_2_). Brain blocks were placed on the chilled stage of a vibratome (Microm), and 200-μm-thick coronal slices containing the POA were cut. The slices were placed in oxygenated ACSF for at least 1 h at RT. The slices were transferred to the recording chamber and superfused with ACSF. The fluorescent GnRH neurons were identified at 10× and 40× objective magnifications of an upright microscope (BX51WI; Olympus) by brief fluorescent illumination and then viewed and patched under Nomarski differential interference contrast optics. Patch pipettes were pulled from glass capillary tubing (PG52151-4; WPI) on a Flaming/Brown micropipette puller (P-97; Sutter Instruments Co.). The pipette solution was filtered and contained 130 mM KCl, 5 mM NaCl, 0.4 mM CaCl_2_, 1 mM MgCl_2_, 10 mM HEPES, and 1.1 mM EGTA (pH 7.3 with KOH). Gramicidin (Sigma) was first dissolved in dimethylsulfoxide (Sigma) to a concentration of 2.5–5 mg/ml and then diluted in the pipette solution just before use to a final concentration of 2.5–5 μg/ml. The gramicidin-perforated patch recordings were performed using an Axopatch 200B amplifier (Molecular Devices). The tip resistance of the electrode was 4–6 MΩ. In initial experiments, the access resistance was monitored, and the experiments began when the resistance stabilized at 50–90 MΩ. This stabilization typically took 15–20 min after gigaseal formation and always corresponded to the resting membrane potential (RMP) of the cell reaching a stable level below -45 mV. In all subsequent cells, experiments were initiated when the RMP reached a stable level below -45 mV. Spontaneous rupture of the seal was evident by a sudden overshooting of the action potentials to values >0 mV. Changes in membrane potential were sampled using a Digidata 1322A interface (Molecular Devices) connected to a PC. Any GnRH-GFP neuron that exhibited a shift in RMP of >2 mV was considered to have responded. Acquisition and subsequent analysis of the acquired data were performed using Clampex9 software (Molecular Devices). Traces were plotted using Origin7 software (MicroCal Software). All recordings were made at RT.

Similar to the *in vitro* studies on acute brain slices, 2 ng/ml recombinant mouse IL-10 or TTX or BM containing TTX, CNQX, AP-5, and picrotoxin were used to investigate the effect of IL-10 on the activity of GnRH neurons.

### Statistical analysis

Data are expressed as the mean ± SEM. Generally, we applied ANOVA with Tukey *post hoc* to test the significance. Arcsine transformation was performed on data collected as percentages before using ANOVA (Fig. 3*E*; Fig. 5*D1*,*D2*). In case the assumption of the homogeneity of variance and normality were violated for ANOVA, non-parametric Mann–Whitney *U* test was used to test the difference between independent groups (Figs. 2, 3*C*,*D*, 4) (STATISTICA 13.3 for Windows, TIBCO).

**Figure 1.**
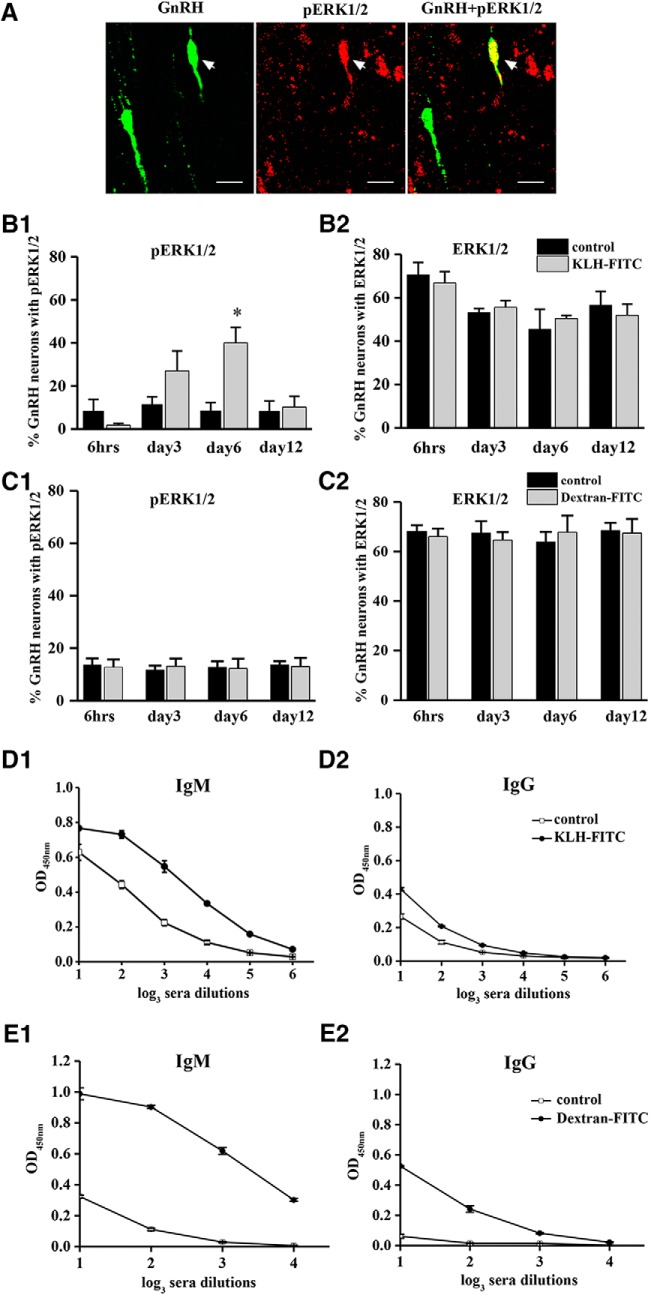
Effect of immunization on ERK1/2 phosphorylation in GnRH neurons. Photomicrographs depict GnRH neurons with pERK1/2 immunoreactivity after 6 d of KLH-FITC immunization. Scale bar: 10 µm (***A***). Histograms show the percentage of ERK1/2- and pERK1/2-immunoreactive GnRH neurons at different time points following KLH-FITC (***B1***, ***B2***) or dextran-FITC (***C1***, ***C2***) injections (*p < 0.05, *n* = 6/group). Antigen-specific serum IgM and IgG levels on day 6 of immunization with KLH-FITC (***D1***, ***D2***) or dextran-FITC (***E1***, ***E2***; *p < 0.05 *n* = 6/group).

**Figure 2.**
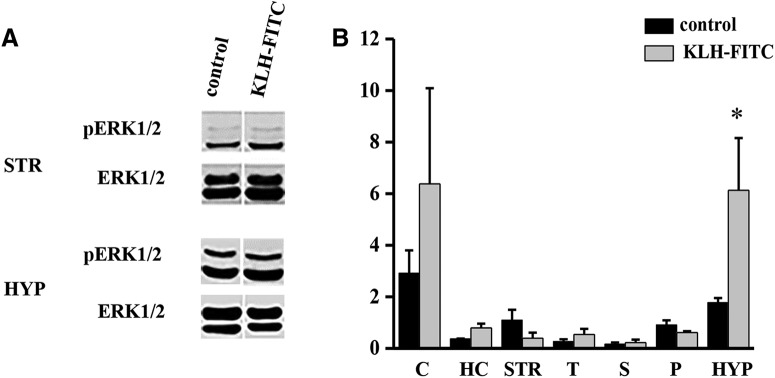
Effect of KLH-FITC on ERK1/2 phosphorylation in different brain regions. Two representative immunoblots probed for pERK1/2 and ERK1/2 from the STR and the HYP of immunized and vehicle-treated mice (***A***). Effect of KLH-FITC on the levels of pERK1/2 in different brain areas shown by Western blot analysis (***B***): quantification of the proportions of pERK1/2 and ERK1/2 in the C, HC, S, T, MS, P, and HYP of control and KLH-FITC-treated animals (*p < 0.05, *n* = 4/group).

**Figure 3.**
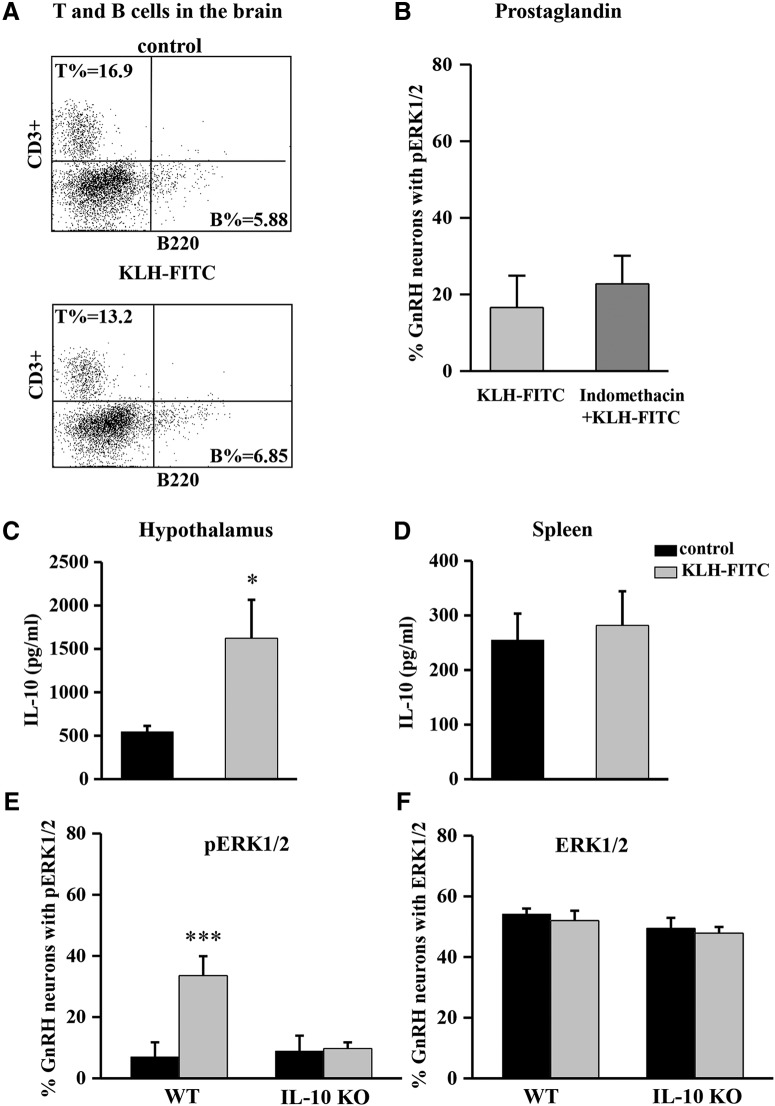
The effect of KLH-FITC on lymphocyte brain infiltration *in vivo* and the role of prostaglandin and IL-10 in KLH-FITC-induced ERK1/2 phosphorylation in GnRH neuron. Representative flow cytometry dot plots of single-cell suspensions from the whole brain of the control and KLH-FITC-injected mice (*n* = 4 pooled samples/group; two separate experiments). Quadrants show CD3 single-positive (T cells), B220 single-positive (B cells), CD3-B220 double-negative, and CD3-B220 double-positive cells (***A***). The histogram shows the percentage of pERK1/2-positive GnRH neurons on day 6 after immunization with KLH-FITC with or without indomethacin treatment (***B***; *n* = 4/group). IL-10 levels are elevated in the HYP (*C*) but not in the spleen (***D***) of KLH-FITC-immunized mice compared to control mice, as shown by ELISA from the HYP and spleen lysates normalized to 100 pg of protein. Data are shown in pg/ml (*p < 0.05, *n* = 5/group). The histogram exhibits the percentage of pERK1/2- (***E***) and ERK-positive GnRH neurons (***F***) in WT, and IL-10 KO mice, comparing KLH-FITC-injected and control mice (***p < 0.001, *n* = 5/group).

**Figure 4.**
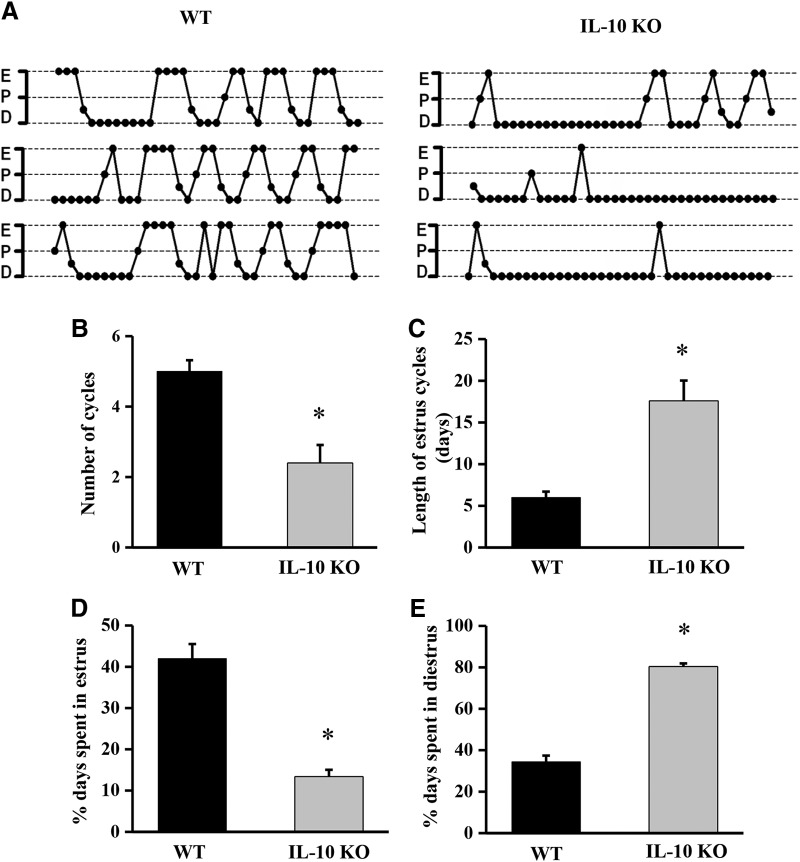
Disrupted estrous cyclicity in IL-10 KO mice. Representative estrous cycle profiles (for individual mice over a five-week period) demonstrating the alterations in estrous cyclicity in WT littermates and IL-10 KO mice (***A***). The graphs show the number of cycles (***B***), cycle length (***C***), and percentage of time spent in the estrus (***D***) and diestrus (***E***) stages for WT and IL-10 KO mice. The histograms show the mean + SEM for each group; *n* = 5, *p < 0.05.

## Results

### KLH-FITC but not dextran-FITC administration induces delayed ERK1/2 phosphorylation in GnRH neurons

Based on antigens and related cytokine production, humoral immune responses can be classified as TD B-cell activation and TI B-cell activation (Defrance et al., 2011; Zubler et al., 2001). In rodent experimental models, the TD and TI immune responses can be effectively dissected using selective immunization protocols such as KLH-FITC and dextran-FITC antigen treatment, respectively (Moreno et al., 1981; Adori et al., 2010). Accordingly, in the first set of experiments, we examined the effects of TD and TI antigen-driven immune challenge on ERK1/2 phosphorylation in GnRH neurons using KLH-FITC and dextran-FITC immunization, respectively.

Immunoreactivity for ERK1/2 and pERK1/2 was detected in GnRH neurons with double-labeling fluorescence immunocytochemistry (Fig. 1*A*). The ERK1/2 immunoreactivity was limited mainly to the cytoplasm of neurons, whereas pERK1/2 immunoreactivity was identified in both nuclear and cytoplasmic compartments. KLH-FITC administration resulted in a significant increase in the number of pERK1/2-immunopositive GnRH neurons on days 6 (Fig. 1*B1*; *n* = 6/group, time: *p* = 0.012, *F* = 4.131, df = 3; treatment: *p* = 0.017, *F* = 6.255, df = 1; interaction of time and treatment: *p* = 0.019, *F* = 3.731, df = 3; two-way ANOVA). KLH-FITC treatment did not change the number of ERK1/2-immunopositive GnRH neurons (Fig. 1*B2* ; *n* = 6/group). The same patterns of ERK1/2 and pERK1/2 expression were observed in GnRH neurons irrespective of their location in the MS, POA, or AH (data not shown). The total number of GnRH neurons varied according to anatomic location (MS: 6 ± 1; POA: 21 ± 1; AH: 5 ± 0.7 GnRH neurons/section) but were not altered by KLH-FITC treatment (data not shown).

In contrast, immunization with a TI antigen, namely, dextran-FITC, failed to have any effect on ERK1/2 phosphorylation in GnRH neurons (Fig. 1*C1*; *n* = 6/group), and the number of GnRH neurons expressing ERK1/2 was not affected by the treatment (Fig. 1*C2* ; *n* = 6/group). The number of GnRH neurons did not change after dextran-FITC treatment (data not shown).

The efficiency of the immunization protocols was tested by measuring whether KLH-FITC or dextran-FITC administration effectively increased the FITC-specific antibody levels in the blood. Six days after KLH-FITC or dextran-FITC injection (for the TD and TI antigens, respectively), the levels of the FITC-specific antibodies of the IgM and IgG isotypes were elevated in the sera of immunized animals compared to the levels in the sera of control animals that received vehicles only (Fig. 1*D1*,*D2*,*E1*,*E2* ).

To examine the area specificity of KLH-FITC-induced pERK1/2, multiple brain regions were micro-dissected or micro-punched, including the C, HC, S, T, MS, P, and HYP, and probed for ERK1/2 and pERK1/2 expression using Western blot analysis 6 d after KLH-FITC treatment. There was no change in pERK1/2 expression in the C (*p* = 1), HC (*p* = 0.31), S (*p* = 1), T (*p* = 0.31), MS (*p* = 0.86), and P (*p* = 0.08; *n* = 4/group; Mann–Whitney; Fig. 2*B*). In contrast, pERK1/2 levels were significantly increased in the HYP of KLH-FITC-immunized animals (*p* = 0.03; Mann–Whitney; Fig. 2*B*). Figure 2*A* shows representative immunoblots probed for ERK1/2 and pERK1/2 expression in the STR and HYP.

### IL-10 mediates KLH-FITC-induced ERK1/2 phosphorylation in GnRH neurons

Next, to understand how ERK1/2 phosphorylation is mediated in GnRH neurons on peripheral immune challenge, potential contributing factors were examined.

Our whole-brain flow cytometry measurements revealed that there was no lymphocyte infiltration into the brain following immunization with the KLH-FITC antigen (*n* = 4 pooled samples/group, two separate experiments; Fig. 3*A*). Administration of indomethacin did not alter the number of pERK1/2-positive GnRH neurons following KLH-FITC treatment on day 6 (*n* = 5/group; Fig. 3*B*), indicating that prostaglandins do not play a role in mediating ERK1/2 phosphorylation in GnRH neurons. The levels of the proinflammatory cytokines IL-1β and TNF-α did not change in the brain following KLH-FITC injection (IL-1β: control ≤ 3 pg/100 mg of tissue, KLH-FITC ≤ 3 pg/100 mg of tissue; TNF-α: control = 13.88 ± 4.94 pg/100 mg of tissue, KLH-FITC = 16.14 ± 2.27 pg/100 mg of tissue; *n* = 5/group).

IL-10 levels were also quantified in the HYPs and spleens of mice following KLH-FITC injection. Interestingly, IL-10 levels were elevated in the HYPs of KLH-FITC-immunized mice compared to the levels in the controls 6 d after treatment (*n* = 4/group, *p* = 0.03, Mann–Whitney; Fig. 3*C*). In contrast, the spleens from the same immune-challenged mice did not show differences in the concentrations of IL-10 (*n* = 4/group; Fig. 3*D*).

To examine whether IL-10 mediates the effect of KLH-FITC on ERK1/2 phosphorylation in GnRH neurons, we examined the expression of pERK1/2 in GnRH neurons 6 d after KLH-FITC immunization in IL-10 KO animals and their WT littermates. Similar to our findings above, the percentage of pERK1/2-positive GnRH neurons increased in WT animals following immunization (*n* = 5/group, WT: control versus KLH-FITC: *p* = 0.0018, *F* = 11.9, df = 1, two-way ANOVA; Fig. 3*E*). However, the KLH-FITC**-**induced ERK1/2 phosphorylation was blocked in IL-10 KO mice (*n* = 5/group, two-way ANOVA; Fig. 3*E*). Expression levels of non-phosphorylated ERK1/2 were not altered by KLH-FITC injection in WT or IL-10 KO mice (Fig. 3*F*). The number of GnRH neurons was not affected in IL-10 KO animals (data not shown).

### Estrous cyclicity is disrupted in IL-10 KO mice

GnRH neurons play a critical role in the regulation of the estrous cycle. To examine whether IL-10 has an effect on the estrous cycle under physiologic conditions, estrous cyclicity was evaluated using vaginal smears for a period of five weeks in IL-10 KO mice and WT littermates (*n* = 5/group; Fig. 4*A*). These data showed that the number of cycles decreased (*p* = 0.016, Mann–Whitney) and the duration of each cycle increased (*p* = 0.012, Mann–Whitney) compared to those in the WT littermates (Fig. 4*B*,*C*). The mutant mice spent significantly less time in the estrus stage (*p* = 0.012, Mann–Whitney) and more time in the diestrus stage (*p* = 0.012, Mann–Whitney) than the WT littermates (Fig. 4*D*,*E*). Representative estrous cycle profiles demonstrating these alterations are presented in Figure 4*A*.

### IL-10 directly alters the electrical activity of GnRH neurons and increases their ERK1/2 phosphorylation

To evaluate the functional relevance of the effect of IL-10 on GnRH neurons, the effect of IL-10 on the electrical activity of GnRH neurons was investigated in GnRH-GFP mice, in which GnRH neurons can be readily identified.

**Figure 5.**
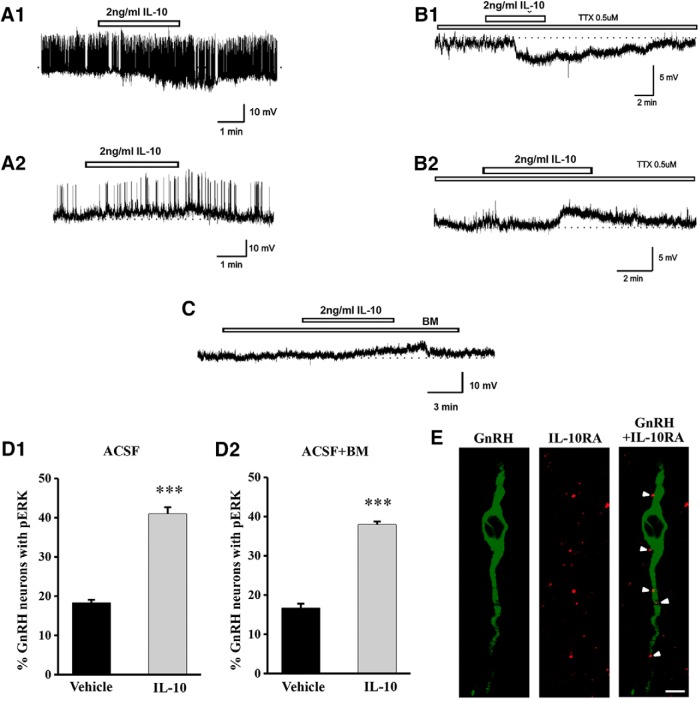
Effect of IL-10 on membrane potential and ERK1/2 phosphorylation of GnRH neurons in acute brain slices. Representative voltage traces from female adult GnRH neurons showing membrane hyperpolarization (***A1***) or depolarization (***A2***) induced by IL-10. The trace depicts membrane depolarization or hyperpolarization in the presence of TTX (***B1***, ***B2***). IL-10-induced depolarization in the presence of BM containing AP-5 (20 μM), CNQX (10 μM), picrotoxin (50 μM) with TTX (0.5 μM; ***C***). Effect of IL-10 on pERK1/2 immunopositivity in GnRH neurons with and without BM (***D1***, ***D2***). Bar graphs show the cumulative percentage of GnRH neurons expressing immunoreactive pERK1/2 in the MS, POA and AH (***p < 0.001, *n* = 6/group). The confocal images show that GnRH neurons (green) express IL-10RA (red, white arrowheads) in the medial septum of female mice (***E***). Scale bar: 10 µm.

Based on the result of the IL-10 ELISA experiments, 2 ng/ml IL-10 was used for the *in vitro* acute brain slice experiments. Bath application of 2 ng/ml IL-10 induced membrane depolarization (mean ± SEM: -2.8 ± 0.2 mV; Fig. 5*A1* ) in 12 (38%) of the 31 GnRH neurons tested from 24 animals, with the remaining neurons exhibiting hyperpolarization (mean ± SEM: -16.5 ± 8 mV; Fig. 5*A2* ; 33%) or being unaffected (29%).

To examine whether IL-10 directly acts on GnRH neurons, we investigated the effect of IL-10 on GnRH neurons in the presence of TTX, a Na^+^-channel blocker. We found that 0.5 μM TTX blocked the action potentials but did not inhibit the IL-10-induced membrane depolarization in 11 of the 28 (39%) GnRH neurons tested (RMP, -71.08 ± 1.52 mV; Fig. 5*B2* ), and membrane hyperpolarization was observed in nine of the 28 GnRH neurons tested from 18 animals (Fig. 5*B1* ). Furthermore, the IL-10-induced depolarization was maintained even in the presence of BM (containing 0.5 μM TTX, 20 μM AP-5, 10 μM CNQX, 50 μM picrotoxin): 13 of 25 (52%) GnRH neurons responded (Fig. 5*C*), while 12 of 25 (48%) were unaffected (25 GnRH neurons from 16 animals).

In addition, to confirm that the IL-10-induced ERK1/2 phosphorylation is the result of direct action of IL-10 on GnRH neurons, we investigated ERK1/2 phosphorylation in GnRH neurons on coapplication of IL-10 and the previously used BM to acute brain slices. The number of GnRH neurons (MS: 6 ± 1; POA: 20 ± 1; AH: 4 ± 1 GnRH neurons/section; data not shown) and the percentage of GnRH neurons containing ERK1/2 immunoreactivity in the slices were similar to those seen in the *in vivo* experiments under basal conditions. Treatment of brain slices with 2 ng/ml IL-10 significantly elevated pERK1/2 expression within GnRH neurons (*n* = 6/group, ACSF: vehicle versus IL-10 treatment: *p* = 0.00017, df = 1, *F* = 376; Fig. 5*D1* ). IL-10-induced ERK1/2 phosphorylation in GnRH neurons was not affected by the blocking cocktail (Fig. 5*D2* ; blocking solution: vehicle versus IL-10 treatment: *p* = 0.00017, df = 1, *F* = 376). The number of GnRH neurons expressing ERK1/2 was not changed by IL-10 treatment (data not shown).

To further evaluate whether GnRH neurons are direct targets of IL-10, we examined IL-10RA immunoreactivity in GnRH neurons (Fig. 5*E*). Confocal laser scanning microscopic examination revealed IL-10RA immunoreactivity on the perikarya and dendrite of GnRH neurons. Using three-dimensional reconstruction our results showed that 26% of all GnRH neurons exhibited IL-10RA immunoreactivity (65 GnRH neurons were tested from two female mice).

## Discussion

We report here that peripheral administration of the TD antigen KLH-FITC induces ERK1/2 phosphorylation in the HYP and in GnRH neurons. This effect showed region specificity as it was absent in C, HC, STR, T, MS, and P. Furthermore, the TI immune response induced by dextran-FITC did not lead to ERK1/2 phosphorylation in either the HYP or in GnRH neurons. While the levels of the proinflammatory cytokines IL-1β and TNFα did not change in the HYPs of immunized mice, the IL-10 levels in the HYPs increased 6 d after KLH-FITC treatment. The role of IL-10 was further confirmed by the fact that the KLH-FITC administration failed to induce ERK1/2 phosphorylation in IL-10 KO mice. Interestingly, the estrous cycle was perturbed in IL-10 KO mice, indicating that IL-10 deficiency could alter the estrous cycle even under pathogen-free, physiologic conditions. The functional relevance of IL-10 was demonstrated by single-cell electrophysiological experiments and *in vitro* experiments on acute brain slices, which showed that IL-10 had a direct effect on electrical activity and ERK1/2 phosphorylation in GnRH neurons. The direct effect of IL-10 on GnRH neurons is further supported by our finding that GnRH neurons express IL-10RA.

Peripheral infections activating innate soluble factors, cells and humoral or cell-mediated adaptive immunity result in reproductive system disease, which, in severe cases, might even lead to infertility (Sheldon et al., 2017; Liu et al., 2018). Several studies have investigated the effect of endotoxin-lipopolysaccharide (LPS) challenge on the reproductive system. Although immune challenge induced by LPS has various effects on the reproductive axis (He et al., 2003; Cardoso et al., 2010; Herman et al., 2013; Izvolskaia et al., 2016), this challenge is a model for a severe, complex, whole-body infection such as septic shock; this model does not mimic the fine-tuned, less severe immune challenges induced by bacteria and viruses. Antibody responses to bacterial or viral antigens require recognition by helper T cells and cooperation between the antigen-specific B cells and T lymphocytes. These antigens are called TD antigens. Many nonprotein antigens, such as polysaccharides and bacterial lipids, stimulate antibody production in the absence of helper T cells, the antigens of which are called TI antigens. Accordingly, the viral/bacterial antigen-induced responses are classified as TD or TI humoral immune responses. Although TD and TI responses are both mediated by cytokines, TI responses do not induce high levels of cytokine production. Based on our immunization protocol (Adori et al., 2010), we used FITC as an antigen conjugated to KLH or dextran to selectively elicit either a TD or TI immune response, respectively. This immunization protocol enabled us to selectively examine the effect of TD and TI antigens with distinct cytokine profiles on the HPG axis.

While the number of pERK1/2immunopositive GnRH neurons significantly increased following KLH-FITC injection, the number of ERK1/2-immunoreactive GnRH neurons was not altered, indicating that the increase is due to phosphorylation of the expressed ERK signal molecules and not due to the alteration of ERK1/2 expression. This increase was absent in dextran-FITC-injected animals, demonstrating that GnRH neurons respond differently to antigens activating distinct immune responses that require cytokine production. Indeed, the levels of IL-10 were significantly elevated in the HYP after KLH-FITC administration, and KLH-FITC-induced ERK1/2 phosphorylation in GnRH neurons was blocked in IL-10 KO mice, indicating the role of IL-10 in mediating GnRH neuron signaling after TD humoral immune responses.

### The direct effect of IL-10 on GnRH neurons

Although it is not clear why the electrical response of GnRH neurons to IL-10 is so heterogeneous, the maintenance of IL-10-induced hyperpolarization/depolarization and pERK1/2 in the presence of BM and TTX clearly demonstrates a direct effect of IL-10 on the postsynaptic neuronal membranes of GnRH neurons. This result is supported by the fact that the GT1-7 mouse hypothalamic GnRH neuronal cell line is regulated by IL-10 (Tu et al., 2007). Previous single-cell microarray studies have suggested that mouse GnRH neurons express IL-10 α and β receptor mRNAs (Liposits, personal communication), providing an effective platform for a direct effect of IL-10 on GnRH neurons. Our IL-10RA-GnRH double immunostaining also confirm the possibility that IL-10 can act directly on GnRH neurons. However, the mechanism of the action of IL-10 on GnRH neurons remains unclear. In single-cell brain slice recordings of dentate gyrus neurons, IL-10 dose-dependently decreases the miniature inhibitory postsynaptic current and the picrotoxin-sensitive tonic current, indicating the existence of pre- and postsynaptic mechanisms (Suryanarayanan et al., 2016). Further studies are required to establish the mechanism of the effect on GnRH neurons. Since IL-10-induced electrical changes were maintained during the blockade of GABAergic and glutamatergic neurotransmission, it seems unlikely that IL-10 was acting via presynaptic modulation of amino acid inputs to GnRH neurons.

The source of the KLH-FITC-induced IL-10 elevation in the HYP is unclear. It is unlikely that IL-10 is derived from the periphery because our IL-10 measurements did not reveal IL-10 elevation in the periphery. It is possible that brain-infiltrating immune cells can produce cytokines such as IL-10 when they are in close proximity of the microglia (Chabot et al., 1999). Our results, however, did not show any difference in the number of brain-infiltrating leukocytes between the control and immunized mice 6 d after KLH-FITC immunization, when the ERK1/2 phosphorylation was most prominent. Changes in the ratio of T and B cell subpopulations, and a consequent functional change, however, cannot be excluded as a possible explanation. An increased proportion of regulatory B and/or T cells might enhance immunosuppression, since regulatory B cells themselves can produce IL-10 (Seifert et al., 2018), while regulatory T cells can shift the microglial polarization toward the M2 phenotype, which is characterized by the production of anti-inflammatory cytokines, including IL-10 production (Zhou et al., 2017). Indeed, activated microglia can secrete IL-10 (Park et al., 2007; Lim et al., 2013; Lobo-Silva et al., 2016). Furthermore, Fujioka et al. (2013) revealed evidence for a close relationship between microglia and GnRH neurons, providing a functional platform for microglia-derived IL-10 activity on GnRH neurons. Based on these findings, we have speculated that IL-10 produced by microglia might alter the functions of GnRH neurons. This theory warrants further investigation.

### The possible functional consequences of IL-10-mediated TD antigen-induced immune challenges in GnRH neurons

One of the most important questions associated with the effect of IL-10 on GnRH and IL-10-mediated TD antigen-induced ERK1/2 phosphorylation is that of the physiologic and clinical relevance.

Understanding the effect of the antibody-mediated immune response on the HPG axis has substantial clinical relevance. Several pathogens that primarily elicit humoral immune responses have an effect on the reproductive system. There is growing evidence to support the role of B lymphocyte-mediated immunity in several intracellular infections, including *Chlamydia trachomatis* (Li and McSorley, 2015). *Chlamydia* infections can cause severe fertility problems, such as pelvic inflammatory disease and involuntary infertility; therefore, *Chlamydia* infection is a serious threat to the health of women worldwide (Li and McSorley, 2015).

Clinical studies have demonstrated that as one of the major immunoregulatory cytokines, IL-10 is beneficial for a successful pregnancy and may increase fertility and fecundity (Wegmann et al., 1993; van Dunné et al., 2006; Cheng and Sharma, 2015). Decreased production of IL-10 is associated with pregnancy loss and increased preeclampsia (Hennessy et al., 1999). Although clinical studies have demonstrated the importance of IL-10 in the periphery, our results suggest for the first time that IL-10 directly alters the function of the central processing unit of fertility and fecundity, i.e., the GnRH neurons. Importantly, the estrous cycle was perturbed in IL-10 KO mice, suggesting an important role for IL-10 in the regulation of the HPG axis. Although the effect of IL-10 on the periphery cannot be ruled out completely, these data suggest that the action of IL-10 on GnRH can be important for regulation of the estrous cycle.

In summary, we report here that the TD humoral immune response results in delayed phosphorylation of ERK1/2 in GnRH neurons due to the effect of IL-10. These studies provide the first evidence of the direct action of IL-10 on GnRH neurons and its potential role in the maintenance of the integrity of the estrous cycle.

## Synthesis

Reviewing Editor: Margaret McCarthy, Univ of Maryland School of Medicine Decisions are customarily a result of the Reviewing Editor and the peer reviewers coming together and discussing their recommendations until a consensus is reached. When revisions are invited, a fact-based synthesis statement explaining their decision and outlining what is needed to prepare a revision will be listed below. The following reviewer(s) agreed to reveal their identity: Sabrina Burmeister.

GnRH neurons are at the apex of control of the reproductive axis and they are known to be influenced by an array of factors. In this study, the authors elucidate a role for immune responses, via interleukin-10, to modifying the physiology of GnRH neurons and disruption of the estrous cycle. A particular strength of the study was the use of multiple levels of analysis from the level of cells to systems. Direct effects of anti-inflammatory IL10 in GnRH neurons have not been shown. Also, brain integration of immune-reproductive system is potentially very important.

Suggestions for improvement

Statistics

1) The authors state that they use Mann-Whitney U tests for two groups and two-way ANOVA for groups of 4. However, they do not explain this choice. Mann-Whitney U is a non-parametric test whereas the ANOVA is parametric. The use of one type versus another needs to be justified. Further, it is not clear to me that this is actually what they did because they report ANOVA results for fig. 4, which is only two groups. Overall, the statistics are unclear.

2) Much of the data is collected as proportions or percentages, which cannot be used directly in parametric tests. It might be suitable to handle this using an arcsine transformation first. Similarly, it would not be appropriate to use ANOVA on the number of cycles (fig. 4). 3

3) Whenever statistics are reported, it would be helpful to know what the factors were and whether an interaction was examined. Statistics (e.g., F statistic) and degrees of freedom should also be included so that the reader can evaluate whether the test was conducted properly.

4) In addition to clarifying the statistics, and perhaps reanalyzing some of the data, the authors should consider how the data are described/reported in the manuscript. It was unclear to me why one would report the mean data for groups when they show the same data in a figure (e.g., 317-321 but this occurred throughout the MS). The additional text was distracting.

5) Also, please always report the statistic and degrees of freedom when reporting a p value. 6

6) When p values are reported in the text, why were asterisks used? E.g., line 384, 394, etc.

General Comments

1. A better description of the mouse models is necessary. Are GnRH-GFP and IL-KO mice commercially available? Have they been validated? If so, publications should be cited.

2. In the experimental design, mice are treated with TD or TI antigen using different route of administration, i.e. subcutaneous or iv. Please, explain.

3. Lines 103-106, brains were microdissected to collect specific sites. Please, add more details to the methods. Microdissection, punches? What landmarks were used for anatomical references for each region. Unilateral?

4. Brain slices for electrophysiology, page 11. Experimental details are missing. Are these males, females? What age? If females, did authors control for estrous cycle stage? These are important information considering the cellular responses obtained (depolarizing and hyperpolarizing).

5. How many animals were used in the ephys experiments? Authors report number of cells but number of mice is also important to ensure reproducibility.

6. It would be useful to have number of mice in all data set, not only in figure legends. The number of mice used in each experiment is very low. Were the experiments replicated or performed in only one cohort? Justification is necessary. Number of mice may also account for the high variability observed in some brain areas illustrated in Figure 2.

7. Data on IL10-KO mice is very interesting but it may not be used as a direct evidence for IL10 effect in GnRH cells. More information could be obtained from those mice. Negative/positive feedback of estrogen, Kisspeptin response and levels are few examples.

8. Line 395: "number and duration of complete cycles greatly decreased..." Authors probably mean number of cycles decreased and duration of each cycle increased.

9. Line 419: " in 9 of 8 GnRH neurons tested " Please, confirm. 8 in 9 GnRH neurons tested?

10. The authors did a good job interpreting their data and developing a story without being too speculative (e.g., lines 503-522). Yet, the authors might want to reconsider the use of the word "assumption" in line 522 as this is more of a conjecture they are making.

11. There are no concerns about the treatment of the animals, but the authors should include reference to an approved IACUC (or similar) protocol. (Line 76)
